# 1-[(6-Chloro­pyridin-3-yl)meth­yl]­imidazolidin-2-one

**DOI:** 10.1107/S1600536812023537

**Published:** 2012-05-31

**Authors:** Rajni Kant, Vivek K. Gupta, Kamini Kapoor, Chetan S. Shripanavar, Kaushik Banerjee

**Affiliations:** a X-ray Crystallography Laboratory, Post-Graduate Department of Physics and Electronics, University of Jammu, Jammu Tawi 180 006, India; b Department of Chemistry, Shivaji University, Kolhapur 416 004, India; c National Research Centre for Grapes, Pune 412 307, India

## Abstract

In the title mol­ecule, C_9_H_10_ClN_3_O, the dihedral angle between the pyridine ring and imidazoline ring mean plane [maximum deviation = 0.031–(3) Å] is 76.2 (1)°. In the crystal, N—H⋯O hydrogen bonds link pairs of mol­ecules to form inversion dimers. In addition, weak C—H⋯N hydrogen bonds and π–π stacking inter­actions between pyridine rings [centroid–centroid distance = 3.977 (2) Å] are observed.

## Related literature

For the background to the insecticidal applications of imidacloprid (*N*-{1-[(6-chloro-3-pyrid­yl)meth­yl]-4,5-dihydro­imidazol-2-yl}nitramide), see: Samaritoni *et al.* (2003 ▶); Suchail *et al.* (2001 ▶, 2004 ▶); Schulz-Jander & Casida (2002 ▶); Kagabu *et al.* (2007 ▶); Pandey *et al.* (2009 ▶). For related structures, see: Kapoor *et al.* (2011 ▶, 2012 ▶); Kant *et al.* (2012 ▶).
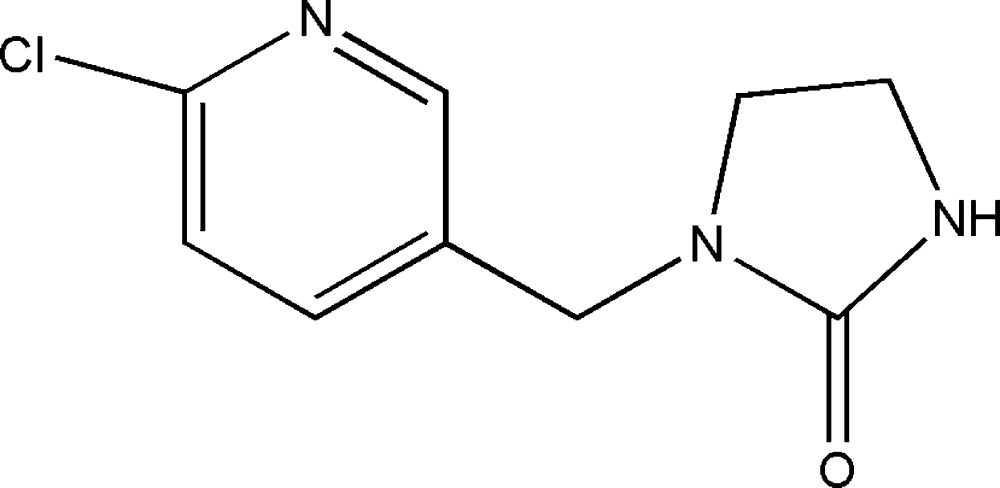


## Experimental

### 

#### Crystal data

C_9_H_10_ClN_3_O
*M* *_r_* = 211.65
Triclinic, 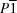

*a* = 5.9864 (3) Å
*b* = 7.4724 (5) Å
*c* = 11.0235 (8) Å
α = 83.103 (6)°
β = 80.040 (5)°
γ = 80.020 (5)°
*V* = 476.26 (5) Å^3^
*Z* = 2
Mo *K*α radiation
μ = 0.37 mm^−1^
*T* = 293 K
0.3 × 0.2 × 0.1 mm

#### Data collection

Oxford Diffraction Xcalibur Sapphire3 diffractometer
Absorption correction: multi-scan (*CrysAlis PRO*; Oxford Diffraction, 2010 ▶) *T*_min_ = 0.835, *T*_max_ = 1.000
6991 measured reflections
1876 independent reflections
1127 reflections with *I* > 2σ(*I*)
*R*_int_ = 0.053

#### Refinement

*R*[*F* ^2^ > 2σ(*F* ^2^)] = 0.068
*wR*(*F* ^2^) = 0.205
*S* = 0.98
1876 reflections
131 parameters
H atoms treated by a mixture of independent and constrained refinement
Δρ_max_ = 0.60 e Å^−3^
Δρ_min_ = −0.26 e Å^−3^

### 

Data collection: *CrysAlis PRO* (Oxford Diffraction, 2010 ▶); cell refinement: *CrysAlis PRO*; data reduction: *CrysAlis PRO*; program(s) used to solve structure: *SHELXS97* (Sheldrick, 2008 ▶); program(s) used to refine structure: *SHELXL97* (Sheldrick, 2008 ▶); molecular graphics: *ORTEP-3* (Farrugia, 1997 ▶); software used to prepare material for publication: *PLATON* (Spek, 2009 ▶).

## Supplementary Material

Crystal structure: contains datablock(s) I, global. DOI: 10.1107/S1600536812023537/lh5478sup1.cif

Structure factors: contains datablock(s) I. DOI: 10.1107/S1600536812023537/lh5478Isup2.hkl

Supplementary material file. DOI: 10.1107/S1600536812023537/lh5478Isup3.cml

Additional supplementary materials:  crystallographic information; 3D view; checkCIF report

## Figures and Tables

**Table 1:** Hydrogen-bond geometry (Å, °)

*D*—H⋯*A*	*D*—H	H⋯*A*	*D*⋯*A*	*D*—H⋯*A*
N11—H11⋯O12^i^	0.85 (5)	2.08 (5)	2.924 (4)	174 (5)
C4—H4⋯N1^ii^	0.93	2.55	3.369 (5)	147

Symmetry codes: (i) 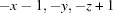; (ii) 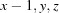.

**Fig. 1.**
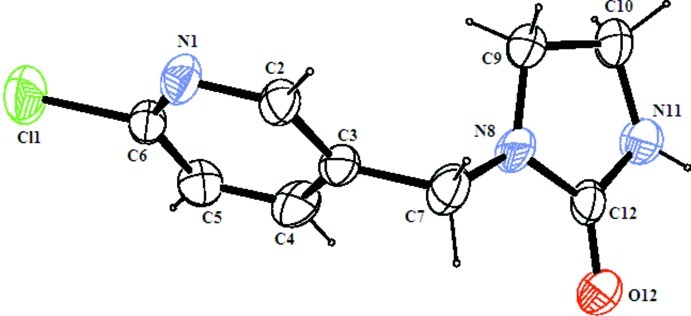
The molecular structure of (I) with thermal ellipsoids drawn at the 40% probability level. H atoms are shown as small spheres of arbitrary radii.

**Fig. 2.**
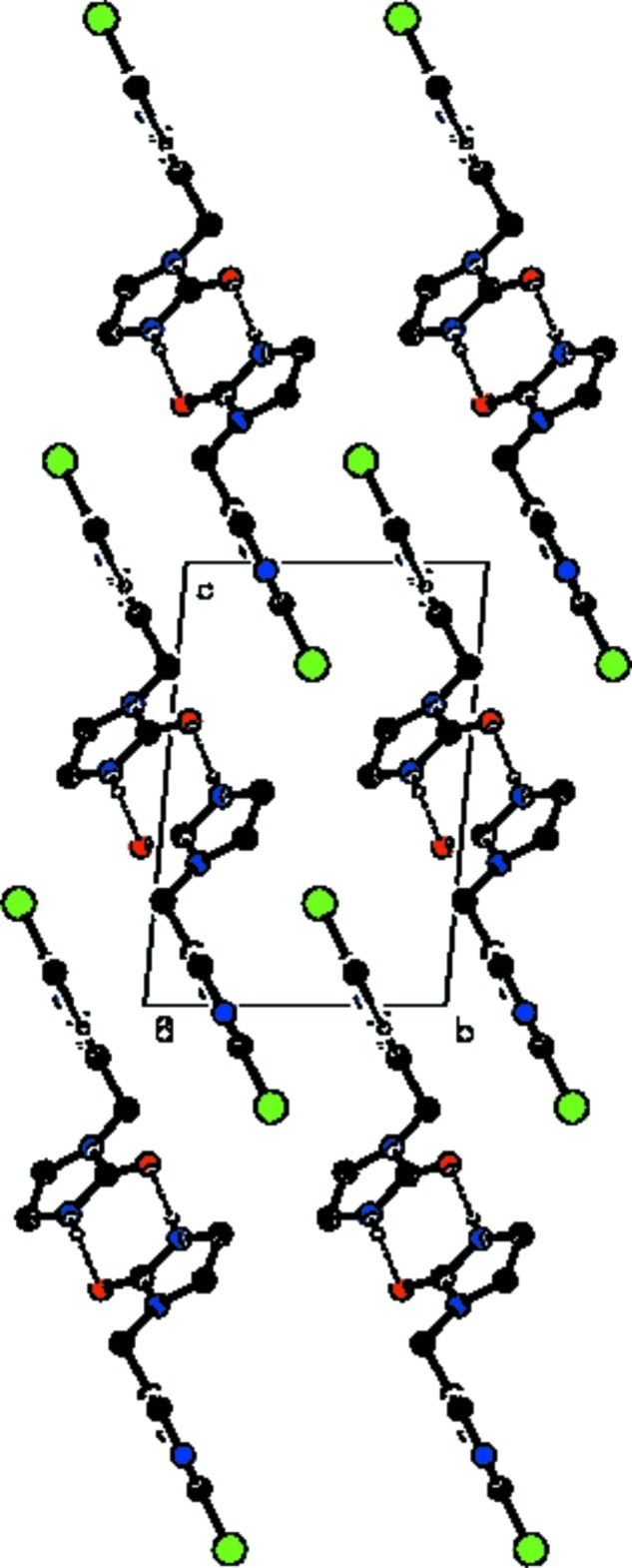
The packing of (I) with broken lines to show N—H···O hydrogen bonds.

## supplementary crystallographic information

### Comment

Imidacloprid is an insecticide which acts as an agonist of the acetylcholine receptor of insect nervous system. Oral, acute and chronic toxicity of imidacloprid and its main metabolite 1-[(6-chloropyridin-3-yl)methyl] imidazolidin-2-one (urea derivative) in mid-gut and rectum were investigated in Apis mellifera (Suchail *et al.*, 2001; Suchail *et al.*, 2004). Acute intoxication by imidacloprid or its metabolites results in rapid appearance of neurotoxicity symptoms, such as hyper-responsiveness and, hyperactivity (Suchail *et al.*, 2001). Many metabolites of imidacloprid have been identified, but the enzymatic basis for their formation has not been reported in many cases (Schulz-Jander & Casida, 2002). Imidacloprid is degraded by liver enzymes to other nitroimines such as the corresponding guanidine and urea derivatives. The fate of imidacloprid in soil environment in terms of the metabolites toxic to vertebrates has been reported by Pandey *et al.* (2009). The supreme biological profile of imidacloprid is giving impulse to the development of new products by modifying the structural features of the prototype (Kagabu *et al.*, 2007). Therefore, in a search for new neonicotinoid insecticides with improved profiles, some neonicotinoid derivatives have been designed and synthesized. The crystal structure of the title compound (I) is shown in Fig. 1.

The bond lengths and angles in (I) show normal values and are comparable with related structures (Kapoor *et al.*, 2011,2012; Kant *et al.*, 2012). The plane through the pyridine ring forms dihedral angle of 76.2 (1)Å with the imidazoline ring plane. In the crystal, N—H···O hydrogen bonds link pairs of molecules to form inversion dimers (Fig. 2). These dimers are linked by weak C—H···N interactions. The crystal structure is further stabilized by π–π interactions between the pyridine ring of the molecule at (*x*, *y*, *z*) and the pyridine ring of an inversion related molecule at (1 - *x*, -*y*, -*z*)[centroid separation = 3.977 (2) Å, interplanar spacing = 3.267 Å and centroid shift = 2.267 Å].

### Experimental

Imidacloprid (0.256 g, 0.001 mol) was dissolved in 5 ml methanol and to it 5 ml of 1 N NaOH solution was added. The reaction mixture was refluxed for about 10 hrs on a water bath at 343K and then cooled. The reaction mixture was neutralized with 1 N HCl solution. The neutralized solution was kept standing for slow evaporation until a white transparent crystalline separated out (m.p. 416 K). LC—MS/MS: 212[*M*+H+], 195, 169, 159, 128, 126, 99, 92 m/*z*.

### Refinement

Hydrogen atom H11 was found in a difference map and refined isotropically. All other H atoms were positioned geometrically and were treated as riding on their parent C atoms, with C—H distances of 0.93–0.97 Å and with *U*_iso_(H) = 1.2*U*_eq_(C).

### Crystal data

**Table:**

C_9_H_10_ClN_3_O	*Z* = 2
*M**_r_* = 211.65	*F*(000) = 220
Triclinic, *P*1	*D*_x_ = 1.476 Mg m^−^^3^
Hall symbol: -P 1	Melting point: 416 K
*a* = 5.9864 (3) Å	Mo *K*α radiation, λ = 0.71073 Å
*b* = 7.4724 (5) Å	Cell parameters from 2653 reflections
*c* = 11.0235 (8) Å	θ = 3.5–28.9°
α = 83.103 (6)°	µ = 0.37 mm^−^^1^
β = 80.040 (5)°	*T* = 293 K
γ = 80.020 (5)°	Block, white
*V* = 476.26 (5) Å^3^	0.3 × 0.2 × 0.1 mm

### Data collection

**Table:**

Oxford Diffraction Xcalibur Sapphire3 diffractometer	1876 independent reflections
Radiation source: fine-focus sealed tube	1127 reflections with *I* > 2σ(*I*)
Graphite monochromator	*R*_int_ = 0.053
Detector resolution: 16.1049 pixels mm^-1^	θ_max_ = 26.0°, θ_min_ = 3.5°
ω scans	*h* = −7→7
Absorption correction: multi-scan (*CrysAlis PRO*; Oxford Diffraction, 2010)	*k* = −9→9
*T*_min_ = 0.835, *T*_max_ = 1.000	*l* = −13→13
6991 measured reflections	

### Refinement

**Table:**

Refinement on *F*^2^	Primary atom site location: structure-invariant direct methods
Least-squares matrix: full	Secondary atom site location: difference Fourier map
*R*[*F*^2^ > 2σ(*F*^2^)] = 0.068	Hydrogen site location: inferred from neighbouring sites
*wR*(*F*^2^) = 0.205	H atoms treated by a mixture of independent and constrained refinement
*S* = 0.98	*w* = 1/[σ^2^(*F*_o_^2^) + (0.1095*P*)^2^] where *P* = (*F*_o_^2^ + 2*F*_c_^2^)/3
1876 reflections	(Δ/σ)_max_ = 0.001
131 parameters	Δρ_max_ = 0.60 e Å^−^^3^
0 restraints	Δρ_min_ = −0.26 e Å^−^^3^

### Special details

**Table:**

Experimental. *CrysAlis PRO*, Oxford Diffraction Ltd., Version 1.171.34.40 (release 27–08-2010 CrysAlis171. NET) (compiled Aug 27 2010,11:50:40) Empirical absorption correction using spherical harmonics, implemented in SCALE3 ABSPACK scaling algorithm.
Geometry. All e.s.d.'s (except the e.s.d. in the dihedral angle between two l.s. planes) are estimated using the full covariance matrix. The cell e.s.d.'s are taken into account individually in the estimation of e.s.d.'s in distances, angles and torsion angles; correlations between e.s.d.'s in cell parameters are only used when they are defined by crystal symmetry. An approximate (isotropic) treatment of cell e.s.d.'s is used for estimating e.s.d.'s involving l.s. planes.
Refinement. Refinement of *F*^2^ against ALL reflections. The weighted *R*-factor *wR* and goodness of fit *S* are based on *F*^2^, conventional *R*-factors *R* are based on *F*, with *F* set to zero for negative *F*^2^. The threshold expression of *F*^2^ > σ(*F*^2^) is used only for calculating *R*-factors(gt) *etc*. and is not relevant to the choice of reflections for refinement. *R*-factors based on *F*^2^ are statistically about twice as large as those based on *F*, and *R*- factors based on ALL data will be even larger.

### Fractional atomic coordinates and isotropic or equivalent isotropic displacement parameters (Å^2^)

**Table:**

	*x*	*y*	*z*	*U*_iso_*/*U*_eq_	
Cl1	0.4849 (2)	0.45046 (14)	−0.23011 (9)	0.0729 (5)	
N1	0.5214 (5)	0.2659 (4)	−0.0175 (3)	0.0489 (8)	
C4	0.0675 (6)	0.2139 (5)	0.0355 (4)	0.0542 (10)	
H4	−0.0858	0.1979	0.0528	0.065*	
C3	0.2141 (5)	0.1420 (4)	0.1179 (3)	0.0410 (8)	
C2	0.4371 (5)	0.1719 (5)	0.0871 (3)	0.0432 (8)	
H2	0.5374	0.1237	0.1427	0.052*	
C5	0.1490 (6)	0.3105 (5)	−0.0738 (3)	0.0496 (9)	
H5	0.0535	0.3601	−0.1316	0.059*	
C6	0.3766 (6)	0.3299 (4)	−0.0931 (3)	0.0436 (8)	
C7	0.1389 (6)	0.0288 (5)	0.2357 (4)	0.0528 (9)	
H7A	0.0729	−0.0717	0.2158	0.063*	
H7B	0.2721	−0.0224	0.2747	0.063*	
N8	−0.0276 (5)	0.1334 (4)	0.3217 (3)	0.0539 (8)	
C9	0.0273 (6)	0.2816 (5)	0.3778 (3)	0.0489 (9)	
H9A	0.0401	0.3877	0.3185	0.059*	
H9B	0.1703	0.2460	0.4108	0.059*	
C10	−0.1757 (6)	0.3212 (5)	0.4818 (4)	0.0556 (10)	
H10A	−0.1250	0.3054	0.5619	0.067*	
H10B	−0.2575	0.4442	0.4688	0.067*	
N11	−0.3167 (6)	0.1876 (5)	0.4724 (3)	0.0645 (10)	
C12	−0.2227 (6)	0.0748 (5)	0.3831 (3)	0.0456 (9)	
O12	−0.2977 (4)	−0.0596 (4)	0.3591 (2)	0.0627 (8)	
H11	−0.435 (8)	0.156 (6)	0.518 (4)	0.091 (15)*	

### Atomic displacement parameters (Å^2^)

**Table:**

	*U*^11^	*U*^22^	*U*^33^	*U*^12^	*U*^13^	*U*^23^
Cl1	0.0942 (9)	0.0714 (8)	0.0479 (7)	−0.0244 (6)	0.0061 (6)	0.0070 (5)
N1	0.0365 (16)	0.0625 (18)	0.0457 (19)	−0.0145 (13)	0.0013 (14)	0.0024 (15)
C4	0.0285 (17)	0.068 (2)	0.068 (3)	−0.0144 (16)	−0.0060 (17)	−0.005 (2)
C3	0.0331 (17)	0.0449 (17)	0.044 (2)	−0.0102 (14)	0.0038 (15)	−0.0095 (15)
C2	0.0330 (17)	0.056 (2)	0.040 (2)	−0.0110 (14)	−0.0023 (15)	−0.0025 (16)
C5	0.043 (2)	0.058 (2)	0.048 (2)	−0.0045 (16)	−0.0123 (17)	−0.0026 (18)
C6	0.048 (2)	0.0440 (18)	0.036 (2)	−0.0116 (15)	0.0023 (16)	−0.0025 (15)
C7	0.048 (2)	0.052 (2)	0.053 (2)	−0.0131 (16)	0.0096 (18)	−0.0017 (18)
N8	0.0459 (16)	0.0637 (19)	0.051 (2)	−0.0266 (14)	0.0192 (14)	−0.0135 (16)
C9	0.051 (2)	0.052 (2)	0.043 (2)	−0.0176 (16)	0.0032 (17)	−0.0041 (17)
C10	0.060 (2)	0.063 (2)	0.040 (2)	−0.0163 (18)	0.0084 (18)	−0.0049 (18)
N11	0.054 (2)	0.090 (2)	0.052 (2)	−0.0363 (19)	0.0183 (17)	−0.0188 (19)
C12	0.0399 (18)	0.064 (2)	0.034 (2)	−0.0194 (16)	−0.0016 (15)	0.0038 (17)
O12	0.0553 (16)	0.0826 (19)	0.0558 (18)	−0.0392 (14)	0.0077 (13)	−0.0107 (15)

### Geometric parameters (Å, º)

**Table:**

Cl1—C6	1.746 (3)	C7—H7B	0.9700
N1—C6	1.298 (4)	N8—C12	1.359 (4)
N1—C2	1.344 (4)	N8—C9	1.442 (4)
C4—C3	1.371 (5)	C9—C10	1.534 (5)
C4—C5	1.386 (5)	C9—H9A	0.9700
C4—H4	0.9300	C9—H9B	0.9700
C3—C2	1.370 (4)	C10—N11	1.437 (5)
C3—C7	1.507 (5)	C10—H10A	0.9700
C2—H2	0.9300	C10—H10B	0.9700
C5—C6	1.373 (5)	N11—C12	1.357 (5)
C5—H5	0.9300	N11—H11	0.85 (4)
C7—N8	1.444 (5)	C12—O12	1.241 (4)
C7—H7A	0.9700		
			
C6—N1—C2	115.8 (3)	C12—N8—C9	111.8 (3)
C3—C4—C5	119.6 (3)	C12—N8—C7	123.6 (3)
C3—C4—H4	120.2	C9—N8—C7	122.2 (3)
C5—C4—H4	120.2	N8—C9—C10	103.9 (3)
C2—C3—C4	117.2 (3)	N8—C9—H9A	111.0
C2—C3—C7	120.5 (3)	C10—C9—H9A	111.0
C4—C3—C7	122.3 (3)	N8—C9—H9B	111.0
N1—C2—C3	124.7 (3)	C10—C9—H9B	111.0
N1—C2—H2	117.6	H9A—C9—H9B	109.0
C3—C2—H2	117.6	N11—C10—C9	102.9 (3)
C6—C5—C4	117.1 (3)	N11—C10—H10A	111.2
C6—C5—H5	121.4	C9—C10—H10A	111.2
C4—C5—H5	121.4	N11—C10—H10B	111.2
N1—C6—C5	125.5 (3)	C9—C10—H10B	111.2
N1—C6—Cl1	116.2 (3)	H10A—C10—H10B	109.1
C5—C6—Cl1	118.3 (3)	C12—N11—C10	112.8 (3)
N8—C7—C3	112.5 (3)	C12—N11—H11	114 (3)
N8—C7—H7A	109.1	C10—N11—H11	133 (3)
C3—C7—H7A	109.1	O12—C12—N11	127.2 (3)
N8—C7—H7B	109.1	O12—C12—N8	124.5 (3)
C3—C7—H7B	109.1	N11—C12—N8	108.3 (3)
H7A—C7—H7B	107.8		
			
C5—C4—C3—C2	0.6 (5)	C3—C7—N8—C12	134.0 (3)
C5—C4—C3—C7	−177.3 (3)	C3—C7—N8—C9	−64.8 (4)
C6—N1—C2—C3	−0.5 (5)	C12—N8—C9—C10	−4.1 (4)
C4—C3—C2—N1	−0.1 (5)	C7—N8—C9—C10	−167.3 (3)
C7—C3—C2—N1	177.8 (3)	N8—C9—C10—N11	1.1 (4)
C3—C4—C5—C6	−0.4 (5)	C9—C10—N11—C12	2.2 (4)
C2—N1—C6—C5	0.6 (5)	C10—N11—C12—O12	175.2 (4)
C2—N1—C6—Cl1	−179.4 (2)	C10—N11—C12—N8	−4.9 (4)
C4—C5—C6—N1	−0.2 (5)	C9—N8—C12—O12	−174.4 (3)
C4—C5—C6—Cl1	179.8 (3)	C7—N8—C12—O12	−11.5 (6)
C2—C3—C7—N8	115.6 (3)	C9—N8—C12—N11	5.7 (4)
C4—C3—C7—N8	−66.6 (4)	C7—N8—C12—N11	168.6 (3)

### Hydrogen-bond geometry (Å, º)

**Table:**

*D*—H···*A*	*D*—H	H···*A*	*D*···*A*	*D*—H···*A*
N11—H11···O12^i^	0.85 (5)	2.08 (5)	2.924 (4)	174 (5)
C4—H4···N1^ii^	0.93	2.55	3.369 (5)	147

Symmetry codes: (i) −*x*−1, −*y*, −*z*+1; (ii) *x*−1, *y*, *z*.
